# Artificial intelligence, data sharing, and privacy for retinal imaging under Brazilian Data Protection Law

**DOI:** 10.1186/s40942-024-00596-8

**Published:** 2025-04-08

**Authors:** Luis Filipe Nakayama, Lucas Zago Ribeiro, Fernando Korn Malerbi, Caio Saito Regatieri

**Affiliations:** Department of Ophthalmology, São Paulo Federal University, St., 821, Vila Clementino, São Paulo, 04023-062 Brazil

**Keywords:** Artificial intelligence, Datasets, Retinal imaging

## Abstract

The integration of artificial intelligence (AI) in healthcare has revolutionized various medical domains, including radiology, intensive care, and ophthalmology. However, the increasing reliance on AI-driven systems raises concerns about bias, particularly when models are trained on non-representative data, leading to skewed outcomes that disproportionately affect minority groups. Addressing bias is essential for ensuring equitable healthcare, necessitating the development and validation of AI models within specific populations. This viewpoint paper explores the critical role of data in AI development, emphasizing the importance of creating representative datasets to mitigate disparities. It discusses the challenges of data bias, the need for local validation of AI algorithms, and the misconceptions surrounding retinal imaging in ophthalmology. Additionally, highlights the significance of publicly available datasets in research and education, particularly the underrepresentation of low- and middle-income countries in such datasets. The Brazilian General Data Protection Law is also examined, focusing on its implications for research and data sharing, including the legal and ethical measures required to safeguard data integrity and privacy. Finally, the manuscript underscores the importance of adhering to the FAIR principles (Findability, Accessibility, Interoperability, and Reusability) to enhance data usability and support responsible AI development in healthcare.

Over recent years, artificial intelligence (AI) algorithms have been increasingly designed and implemented in various facets of daily activities. Notably, the healthcare sector has emerged as one of the main focus of research and investment, where AI-enabled systems have made significant strides across diverse domains, such as radiology, and intensive care, with ophthalmology as a promising field [1–4]. This paper explores the issue of bias in AI within healthcare, emphasizing the importance of representative datasets, ethical data sharing, and global collaboration to ensure the development of responsible and equitable AI technologies.

## Bias in artificial intelligence for healthcare

In healthcare, the utilization of AI models trained on non-representative data can lead to dangerous outcomes, with potentially skewed and prejudiced results that disproportionately affect minority groups [5–7]. Bias in healthcare AI is an intricate, multifaceted, demanding, concerted effort to mitigate these risks effectively [8].

**Fig. 1:**
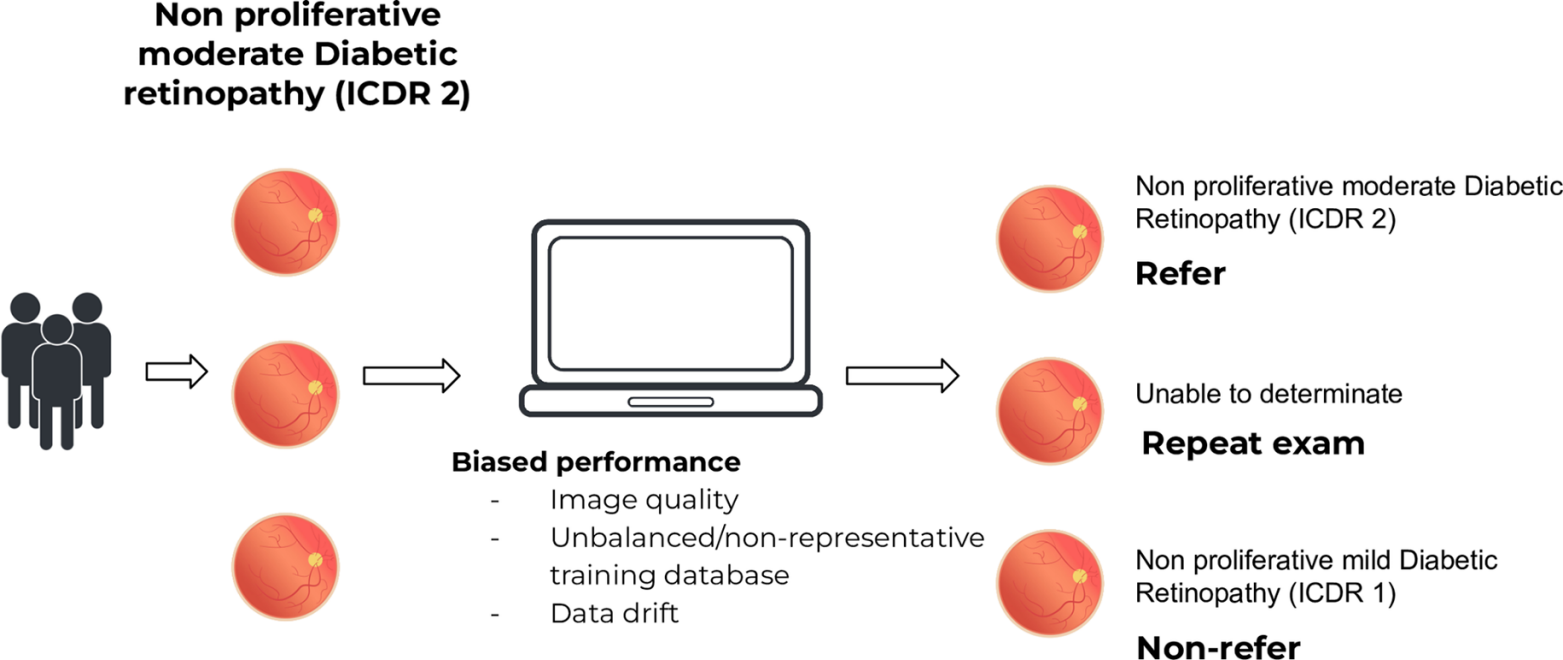
Example of biased outcome in a diabetic retinopathy automatic screening program. Patients with the same disease stage with different outcomes secondary to slightly different classifications

Data bias, deriving from the developing algorithms trained on non-representative datasets, stands out as a critical concern in the field of AI development. Data is the cornerstone upon which AI advancements are built and the description of the included data for algorithm training and of datasets are critical for improving reproducibility and identify possible biases in AI development [9, 10]. The creation and use of representative, well-balanced datasets play a pivotal role in facilitating algorithm development, fostering reproducibility in studies, and enabling local validation processes, all of which are important for reducing disparities among different subpopulations [8, 11]. (Fig. 1)

In evaluating real-world performance, it is important to validate algorithms within the specific target population [12]. Algorithmic high-performance training and validating metrics do not inherently guarantee generalizability. Therefore, before deploying any AI-enabled system, engaging in rigorous local validation efforts is paramount. As an illustrative example, pneumonia screening and diabetic retinopathy algorithms exhibit high algorithmic accuracy but perform poorly when applied to data from external institutions, even when those institutions are within the same country [13, 14].

More examples of AI bias in healthcare highlight the risks of relying on non-representative data. For instance, cardiovascular disease prediction models trained predominantly on populations from high-income countries often fail to account for the genetic, lifestyle, and environmental differences present in Latin American populations. These biases can lead to inaccurate risk assessments, resulting in inappropriate treatments or delayed care. Likewise, AI-driven diagnostic tools for skin conditions frequently struggle to accurately diagnose diseases in patients with darker skin tones [15, 16]. Furthermore, AI algorithms developed for radiology may overlook disease markers that manifest differently in Latin American patients, reinforcing the importance of using local data to ensure these systems work equitably across diverse populations [13].

## Retinal imaging misconceptions

In ophthalmology, there is a popular misconception regarding retinal images and iris images, corroborated by movies and TV series eye scanners [13]. While retinal images are unique to each individual, a specialized camera is needed to take them. Also, the lack of a dataset linking these scans to personal information significantly reduces the risk of reidentification. As a result, the likelihood of uncovering new information about an individual from sharing retinal scans is minimal [17].

## Data sharing and open data

Publicly available datasets play a central role in facilitating various aspects of research, education, and bias assessment, offering a valuable alternative to the considerable costs and challenges associated with developing databases. However, the landscape of publicly accessible datasets predominantly features a representation of high-income countries, with a scarcity of datasets originating from low- and middle-income countries (LMICs) [18–20].

Latin America and Brazil remain underrepresented in publicly available datasets. In Brazil’s specific case, most datasets are sourced from governmental entities such as Brazilian DATASUS and the Institute of Geography and Statistics databases [18]. For ophthalmological datasets, the majority originate from high-income countries (HICs) [19]. Within Brazil, the retinal datasets are from São Paulo and Bahia [21–23] and include only retinal fundus photos modality.

There is a need for greater diversity and inclusivity in dataset availability. Furthermore, international initiatives such as the European Data Governance Act, the United States National Institute of Health, and Dutch ZonMw are actively promoting open science [11, 24, 25]. These efforts hold the promise of fostering collaboration and knowledge exchange, leading to a more comprehensive and inclusive landscape of accessible datasets.

## Brazilian General Data Protection Law

In Brazil, the Brazilian General Data Protection Law (LGPD) is responsible for protecting the rights of privacy and personal data of individuals in Brazil and regulating the processing of personal data by organizations, both domestic and foreign, operating in the country [26]. The legislative process started in 2010, was enacted in August 2018, and came into effect in September 2021. Throughout this period, there were consultations, debates, and revisions to the draft legislation, involving various government agencies, experts, and stakeholders, to ensure that it aligned with international standards and addressed the specific privacy concerns of the Brazilian population.

The LGPD bears similarities to the European General Data Protection Regulation (GDPR) in its objectives and principles. Article 5 defines personal data as any information related to an identified or identifiable natural person. It encompasses identifying information, contact details, biometric data, financial information, and sensitive personal data. Sensitive personal data refers to information related to an individual’s racial or ethnic origin, religious beliefs, political opinions, health data, genetic or biometric data, sexual orientation, or criminal records.

The LGPD establishes clear guidelines regarding the use of personal data for research purposes, emphasizing that such data should exclusively serve the original purpose for which it was collected, particularly in the context of scientific research. Obtaining written consent represents the primary mechanism for the lawful collection and processing of research data.

## Research and data sharing under LGPD

Research groups under the LGPD are organizations devoid of financial interests and dedicated to conducting research with objectives spanning historical, scientific, technological, or statistical domains. These groups are driven by a commitment to both fundamental and applied research, aligning with the overarching goals of advancing knowledge and understanding.

The LGPD articles 7 and 11 establish that in instances related to public health research, researchers may access personal data without direct patient communication and consent, provided that the data is securely stored and pseudonymized. Pseudonymization ensures that individuals’ identities remain safeguarded while allowing valuable research to occur. It also consists of data protection techniques to replace identifiable information with encrypted information, allowing for more secure data analysis.

It’s important to note that de-identified data falls outside the LGPD’s and facilitates compliant data sharing. De-identified data pertains to information that has undergone rigorous processing techniques, rendering it incapable of identifying specific individuals. This practice aligns with the LGPD’s provisions and fosters responsible data utilization for research purposes.

However, it is essential to implement appropriate legal and ethical measures to protect the confidentiality and security of data, regardless of whether it is considered personal or de-identified [11]. Safeguarding the integrity and privacy of data ensures compliance with the LGPD and promotes responsible data handling practices.

## Data usability

Beyond data availability, it is essential to establish a robust infrastructure that fosters the reusability of data, guided by the FAIR principles—Findability, Accessibility, Interoperability, and Reusability [27].

Findability ensures that data can be located swiftly and effectively by both humans and computer systems. Accessibility guarantees that data is readily retrievable and accessible for download or use. Interoperability ensures that data is formatted in a manner that facilitates seamless integration with other datasets. Additionally, data should be meticulously documented and prepared to support its reuse in research, complete with comprehensive metadata and detailed descriptions (Table 1).

**Table 1:** Brazilian publicly available datasets FAIR concepts

	Findability	Accessibility	Interoperability	Reusability
Sistema de Internação Hospitalar [24]	datasus.saude.gov.br	Publicly available	Comma-separated values file with tabular data	Metadata dictionary
Pesquisa Nacional por Amostra de Domicílio [25]	www.ibge.gov.br	Publicly available	HTML, JSON, ODS, XML file with tabular data	Metadata dictionary
BRAX [26]	https://physionet.org/content/brax/1.1.0/	Credentialed access	Comma-separated values file with tabular labels and DICOMs.	Metadata dictionary, GitHub repository
mBRSET [18]	https://physionet.org/content/mbrset/1.0/	Credentialed access	Comma-separated values file with JPEG files.	Metadata dictionary, GitHub repository

## Data standardization

Data standardization is crucial for information exchange, enabling effective communication between various devices and information systems, such as electronic health records (EHRs), imaging devices, and AI systems [28, 29]. This standardization is not only vital for ensuring consistent patient care but also plays a critical role in the development and integration of AI systems.

In the context of ophthalmology, for retinal fundus photography and optical coherence tomography (OCT) exams, the American Academy of Ophthalmology recommends adopting the Digital Imaging and Communications in Medicine (DICOM) format [30]. DICOM is the standard for handling, storing, printing, and transmitting information in medical imaging, which ensures uniformity across different devices and platforms. However, the DICOM format presents several challenges. It lack compatibility across different devices, making it difficult to exchange data between systems. Extracting DICOM files from storage systems can be cumbersome, and the files themselves need to be deidentified to protect patient privacy, as they contain sensitive metadata. Moreover, DICOM is a complex format, which can pose implementation challenges, particularly for smaller healthcare providers or systems with limited technical resources [31].

As an alternative, lossless compression formats have been suggested to encode the same data contained in DICOM files. These formats maintain the integrity of the data while potentially offering more straightforward implementation and compatibility across different platforms [30].

Fast Healthcare Interoperability Resources (FHIR) is a standard for healthcare data exchange developed by Health Level Seven International [32]. It is designed to enable interoperability between different healthcare systems by providing a framework for structuring and sharing data in a way that is both easy to implement and scalable. FHIR plays a critical role in healthcare AI development, as it facilitates the data integration from sources like EHRs, imaging systems, and clinical databases, which are essential for AI models.

While FHIR helps address interoperability challenges, its adoption in LMICs faces hurdles. It requires digital maturity, such as EHR systems and technical expertise, which are often lacking in rural areas. Additionally, FHIR doesn’t automatically ensure data quality, and poor-quality data can still impact AI model performance. Strong data governance, including encryption and authentication, is also necessary to protect sensitive health information when using FHIR.

## Federated learning

Federated learning (FL) is a decentralized approach to machine learning that allows institutions to train AI models collaboratively without sharing data. Instead, only model updates are sent to a central server, addressing privacy concerns in fields like healthcare [33, 34].

One of the major promises of federated learning lies in its ability to leverage diverse datasets across regions and institutions, enabling the development of more generalizable and robust AI models. This could significantly benefit LMICs, where data is often scarce and access to high-quality healthcare datasets is limited. By allowing institutions in LMICs to collaborate without compromising patient privacy, FL opens new possibilities for creating AI systems that reflect the diverse health profiles of these populations.

However, FL faces challenges in LMICs, such as limited infrastructure, including high-speed internet and computational resources [34]. The continuous communication required for FL can strain network bandwidth, and inconsistent data quality may affect model performance. Additionally, although data stays local, FL is still vulnerable to adversarial attacks that could extract sensitive information from model updates [35].

## Discussion

The rising integration of artificial intelligence in healthcare has brought about significant advancements across various medical domains, yet it has also highlighted the critical issue of bias in AI algorithms. Addressing bias is a complex challenge, but one essential solution lies in the sharing of representative datasets. These datasets enable the development and validation of AI models within the specific populations they aim to serve, reducing the risk of biased outcomes.

However, the landscape of publicly available datasets is heavily skewed toward high-income countries, leaving a significant gap in representation, particularly for LMICs [19, 20]. Latin America, including Brazil, faces underrepresentation in available datasets, further emphasizing the need for comprehensive data-sharing efforts [18].

The underrepresentation of LMIC datasets has a direct impact on healthcare outcomes. AI models trained predominantly on data from HICs, where healthcare infrastructure, disease prevalence, and patient demographics differ, may not perform effectively for LMIC populations. For instance, AI models developed for diabetic retinopathy screening using data from North America or Europe may not account for the distinct disease progression patterns observed in Brazilian patients, leading to misdiagnoses or reduced screening efficacy.

In Brazil, these challenges are further compounded by healthcare disparities. Publicly available datasets are mostly derived from urban centers, leaving rural and underserved regions underrepresented. This imbalance exacerbates health inequities, as AI tools deployed across the country may not be adequately validated for use in all regions, leading to inaccurate diagnoses and treatment in remote or low-income areas. Smaller healthcare providers, particularly those serving rural populations, would benefit most from AI models trained on data that reflect their unique patient demographics. These underserved regions, where healthcare systems face significant challenges such as limited access to specialized care and medical infrastructure, present an opportunity for AI to bridge gaps in healthcare delivery. However, the absence of representative data from these regions diminishes AI’s potential in such settings.

To address these issues, improving dataset diversity is essential. Establishing partnerships among government agencies, academic institutions, and healthcare providers could facilitate the development of more inclusive datasets that better represent the full spectrum of patient demographics in Brazil and other LMICs. These collaborations would ensure that data from rural and underserved regions are incorporated, enhancing the generalizability and fairness of AI models.

In addition to diverse datasets, robust legal frameworks are crucial. The enactment of data protection laws like the Brazilian General Data Protection Law aligns with global efforts to safeguard individual privacy rights. The LGPD defines personal data, outlines guidelines for research data usage, and emphasizes the importance of obtaining written consent for lawful data collection. It also recognizes the significance of pseudonymization in enabling secure access to data for public health research while protecting individuals’ identities.

Improving data-sharing practices is equally important. Establishing secure, ethical systems for data exchange will accelerate AI advancements in healthcare. Brazil should participate in international open science initiatives, adopting global standards for data sharing while adhering to local privacy regulations. Such collaboration would enable broader access to knowledge and contribute to the development of fairer AI technologies.

Future research should focus on validating AI algorithms within the populations they are designed to serve [12]. Assessing the real-world performance of AI models across different Latin American regions is crucial for identifying and mitigating bias. Furthermore, interdisciplinary collaboration among data scientists, healthcare professionals, and legal experts is necessary to address the ethical and technical challenges of AI development, fostering fairness, transparency, and accountability in healthcare systems.

Innovative methods for collecting diverse data in resource-limited settings should also be explored. Mobile health technologies and telemedicine offer opportunities to gather high-quality data from underrepresented regions, such as rural Brazil, improving the inclusivity and performance of AI models.

Finally, the distinction between pseudonymized and de-identified data is crucial and facilitates compliant data sharing. Nonetheless, irrespective of data type, implementing robust legal and ethical measures to safeguard data integrity and privacy remains paramount. These practices ensure alignment with the LGPD and support responsible data use in research and beyond. A coordinated effort by governments, academia, and private entities will be vital to ensure equitable AI development, ultimately leading to improved healthcare outcomes in Brazil and globally.

## Data Availability

No datasets were generated or analysed during the current study.

## References

[1] Esteva A, Kuprel B, Novoa RA, Ko J, Swetter SM, Blau HM, et al. Dermatologist-level classification of skin cancer with deep neural networks. Nature. 2017;542:115–8. 10.1038/nature21056. 28117445 10.1038/nature21056 PMC8382232

[2] Ipp E, Liljenquist D, Bode B, Shah VN, Silverstein S, Regillo CD, et al. Pivotal evaluation of an artificial intelligence system for autonomous detection of referrable and vision-threatening diabetic retinopathy. JAMA Netw Open. 2021;4:e2134254. 10.1001/jamanetworkopen.2021.34254. 34779843 10.1001/jamanetworkopen.2021.34254 PMC8593763

[3] Muehlematter UJ, Daniore P, Vokinger KN. Approval of artificial intelligence and machine learning-based medical devices in the USA and Europe (2015-20): a comparative analysis. Lancet Digit Health. 2021;3:e195–203. 10.1016/S2589-7500(20)30292-2. 33478929 10.1016/S2589-7500(20)30292-2

[4] Esteva A, Robicquet A, Ramsundar B, Kuleshov V, DePristo M, Chou K, et al. A guide to deep learning in healthcare. Nat Med. 2019;25:24–9. 10.1038/s41591-018-0316-z. 30617335 10.1038/s41591-018-0316-z

[5] Alenichev A, Kingori P, Grietens KP. Reflections before the storm: the AI reproduction of biased imagery in global health visuals. Lancet Glob Health. 2023;11:e1496–8. 10.1016/S2214-109X(23)00329-7. 37572687 10.1016/S2214-109X(23)00329-7

[6] Schramowski P, Turan C, Andersen N, Rothkopf CA, Kersting K. Large pre-trained language models contain human-like biases of what is right and wrong to do. Nat Mach Intell. 2022;4:258–68. 10.1038/s42256-022-00458-8.

[7] Habib AR, Lin AL, Grant RW. The epic Sepsis Model Falls Short—the importance of external validation. JAMA Intern Med. 2021;181:1040–1. 10.1001/jamainternmed.2021.3333. 34152360 10.1001/jamainternmed.2021.3333

[8] Nakayama LF, Matos J, Quion J, Novaes F, Mitchell WG, Mwavu R et al. Unmasking biases and navigating pitfalls in the ophthalmic Artificial Intelligence lifecycle: a review. 2023. 10.48550/ARXIV.2310.04997 10.1371/journal.pdig.0000618 PMC11460710 39378192

[9] Cacciamani GE, Chu TN, Sanford DI, Abreu A, Duddalwar V, Oberai A, et al. PRISMA AI reporting guidelines for systematic reviews and meta-analyses on AI in healthcare. Nat Med. 2023;29:14–5. 10.1038/s41591-022-02139-w. 36646804 10.1038/s41591-022-02139-w

[10] Gebru T, Morgenstern J, Vecchione B, Vaughan JW, Wallach H, Iii HD, et al. Datasheets for datasets. Commun ACM. 2021;64:86–92. 10.1145/3458723.

[11] de Kok JWTM, de la Hoz MÁA, de Jong Y, Brokke V, Elbers PWG, Thoral P, et al. Sci Data. 2023;10:404. 10.1038/s41597-023-02256-2. A guide to sharing open healthcare data under the General Data Protection Regulation. 10.1038/s41597-023-02256-2 PMC10290652 37355751

[12] Youssef A, Pencina M, Thakur A, Zhu T, Clifton D, Shah NH. External validation of AI models in health should be replaced with recurring local validation. Nat Med. 2023;29:2686–7. 10.1038/s41591-023-02540-z. 37853136 10.1038/s41591-023-02540-z

[13] Zech JR, Badgeley MA, Liu M, Costa AB, Titano JJ, Oermann EK. Variable generalization performance of a deep learning model to detect pneumonia in chest radiographs: a cross-sectional study. PLoS Med. 2018;15:e1002683. 10.1371/journal.pmed.1002683. 30399157 10.1371/journal.pmed.1002683 PMC6219764

[14] Lee AY, Yanagihara RT, Lee CS, Blazes M, Jung HC, Chee YE, et al. Multicenter, Head-to-Head, Real-World Validation Study of Seven Automated Artificial Intelligence Diabetic Retinopathy Screening Systems. Diabetes Care. 2021;44:1168–75. 10.2337/dc20-1877. 33402366 10.2337/dc20-1877 PMC8132324

[15] Buolamwini J, Gebru T. Gender Shades: Intersectional Accuracy Disparities in Commercial Gender Classification. In: Friedler SA, Wilson C, editorsFeb. Proceedings of the 1st Conference on Fairness, Accountability and Transparency. PMLR; 23–24 2018. pp. 77–91. Available: https://proceedings.mlr.press/v81/buolamwini18a.html

[16] Adamson AS, Smith A. Machine learning and health care disparities in dermatology. JAMA Dermatol. 2018;154:1247–8. 10.1001/jamadermatol.2018.2348. 30073260 10.1001/jamadermatol.2018.2348

[17] Lum F. Balancing benefits and risks: the case for retinal images to be considered as non-protected health information for research purposes. Ophthalmology. 2024. 10.1016/j.ophtha.2024.07.031. 39127409 10.1016/j.ophtha.2024.07.031

[18] Restrepo D, Quion J, Vásquez-Venegas C, Villanueva C, Anthony Celi L, Nakayama LF. A scoping review of the landscape of health-related open datasets in Latin America. PLOS Digit Health. 2023;2:e0000368. 10.1371/journal.pdig.0000368. 37878549 10.1371/journal.pdig.0000368 PMC10599518

[19] Khan SM, Liu X, Nath S, Korot E, Faes L, Wagner SK, et al. A global review of publicly available datasets for ophthalmological imaging: barriers to access, usability, and generalisability. Lancet Digit Health. 2020. 10.1016/S2589-7500(20)30240-5. 33735069 10.1016/S2589-7500(20)30240-5 PMC7618278

[20] Celi LA, Cellini J, Charpignon M-L, Dee EC, Dernoncourt F, Eber R, et al. Sources of bias in artificial intelligence that perpetuate healthcare disparities—A global review. PLOS Digit Health. 2022;1:e0000022. 10.1371/journal.pdig.0000022. 36812532 10.1371/journal.pdig.0000022 PMC9931338

[21] Nakayama LF, Restrepo D, Matos J, Ribeiro LZ, Malerbi FK, Celi LA, et al. BRSET: a Brazilian Multilabel Ophthalmological dataset of retina fundus photos. PLOS Digit Health. 2024;3:e0000454. 10.1371/journal.pdig.0000454. 38991014 10.1371/journal.pdig.0000454 PMC11239107

[22] Wu C, Restrepo D, Nakayama LF, Ribeiro LZ, Shuai Z, Barboza NS, et al. MBRSET: a portable retina fundus photos benchmark dataset for clinical and demographic prediction. bioRxiv. 2024. 10.1101/2024.07.11.24310293.39803507

[23] Pires R, Jelinek HF, Wainer J, Valle E, Rocha A. Advancing bag-of-visual-words representations for lesion classification in retinal images. PLoS ONE. 2014;9:e96814. 10.1371/journal.pone.0096814. 24886780 10.1371/journal.pone.0096814 PMC4041723

[24] Everything about FAIR data management. In: ZonMw [Internet]. [cited 26 Dec 2023]. Available: https://www.zonmw.nl/en/research-and-results/fair-data-and-data-management/

[25] Kozlov M. NIH issues a seismic mandate: share data publicly. Nature. 2022;602:558–9. 10.1038/d41586-022-00402-1. 35173323 10.1038/d41586-022-00402-1

[26] Lei Geral de Proteção de Dados Pessoais (LGPD). [cited 26 Dec 2023]. Available: https://www.planalto.gov.br/ccivil_03/_ato2015-2018/2018/lei/L13709compilado.htm

[27] Wilkinson MD, Dumontier M, Aalbersberg IJJ, Appleton G, Axton M, Baak A, et al. The FAIR Guiding principles for scientific data management and stewardship. Sci Data. 2016;3:160018. 10.1038/sdata.2016.18. 26978244 10.1038/sdata.2016.18 PMC4792175

[28] Baxter SL, Lee AY. Gaps in standards for integrating artificial intelligence technologies into ophthalmic practice. Curr Opin Ophthalmol. 2021;32:431–8. 10.1097/ICU.0000000000000781. 34231531 10.1097/ICU.0000000000000781 PMC8373825

[29] Halfpenny W, Baxter SL. Towards effective data sharing in ophthalmology: data standardization and data privacy. Curr Opin Ophthalmol. 2022;33:418–24. 10.1097/ICU.0000000000000878. 35819893 10.1097/ICU.0000000000000878 PMC9357189

[30] Lee AY, Campbell JP, Hwang TS, Lum F, Chew EY, American Academy of Ophthalmology. Recommendations for standardization of images in Ophthalmology. Ophthalmology. 2021;128:969–70. 10.1016/j.ophtha.2021.03.003. 33832778 10.1016/j.ophtha.2021.03.003 PMC8335850

[31] Mackenzie A, Lewis E, Loveland J. Successes and challenges in extracting information from DICOM image databases for audit and research. Br J Radiol. 2023;96:20230104. 10.1259/bjr.20230104. 37698251 10.1259/bjr.20230104 PMC10607388

[32] Index - FHIR v5.0.0. [cited 30 Sep 2024]. Available: https://www.hl7.org/fhir/index.html.

[33] Nguyen DC, Pham Q-V, Pathirana PN, Ding M, Seneviratne A, Lin Z, et al. Federated Learning for smart healthcare: A survey. ACM Comput Surv. 2023;55: 1–37. 10.1145/3501296.

[34] Xu J, Glicksberg BS, Su C, Walker P, Bian J, Wang F. Federated Learning for Healthcare Informatics. Int J Healthc Inf Syst Inform. 2021;5: 1–19. 10.1007/s41666-020-00082-4. 10.1007/s41666-020-00082-4 PMC7659898 33204939

[35] Nakayama LF, de Matos JCRG, Stewart IU, Mitchell WG, Martinez-Martin N, Regatieri CVS, et al. Retinal scans and data sharing: The privacy and scientific development equilibrium. Mayo Clinic Proceedings: Digital Health. 2023;1: 67–74. 10.1016/j.mcpdig.2023.02.003. 10.1016/j.mcpdig.2023.02.003 PMC11975763 40206726

